# The effect of chemotherapy on programmed cell death 1/programmed cell death 1 ligand axis: some chemotherapeutical drugs may finally work through immune response

**DOI:** 10.18632/oncotarget.7631

**Published:** 2016-02-23

**Authors:** Min Luo, Liwu Fu

**Affiliations:** ^1^ State Key Laboratory of Oncology in South China, Collaborative Innovation Center for Cancer Medicine, Guangdong Esophageal Cancer Institute, Sun Yat-Sen University Cancer Center, Guangzhou, China

**Keywords:** programmed cell death 1, programmed cell death 1 ligand, chemotherapy, immunotherapy

## Abstract

Most tumors are immunogenic which would trigger some immune response. Chemotherapy also has immune potentiating mechanisms of action. But it is unknown whether the immune response is associated with the efficacy of chemotherapy and the development of chemoresistance. Recently, there is a growing interest in immunotherapy, among which the co-inhibitory molecules, programmed cell death 1/programmed cell death 1 ligand (PD-1/PD-L1) leads to immune evasion. Since some reports showed that conventional chemotherapeutics can induce the expression of PD-L1, we try to summarize the effect of chemotherapy on PD-1/PD-L1 axis and some potential molecules relevant to PD-1/PD-L1 in chemoresistance in this review.

## INTRODUCTION

**Figure 1:**
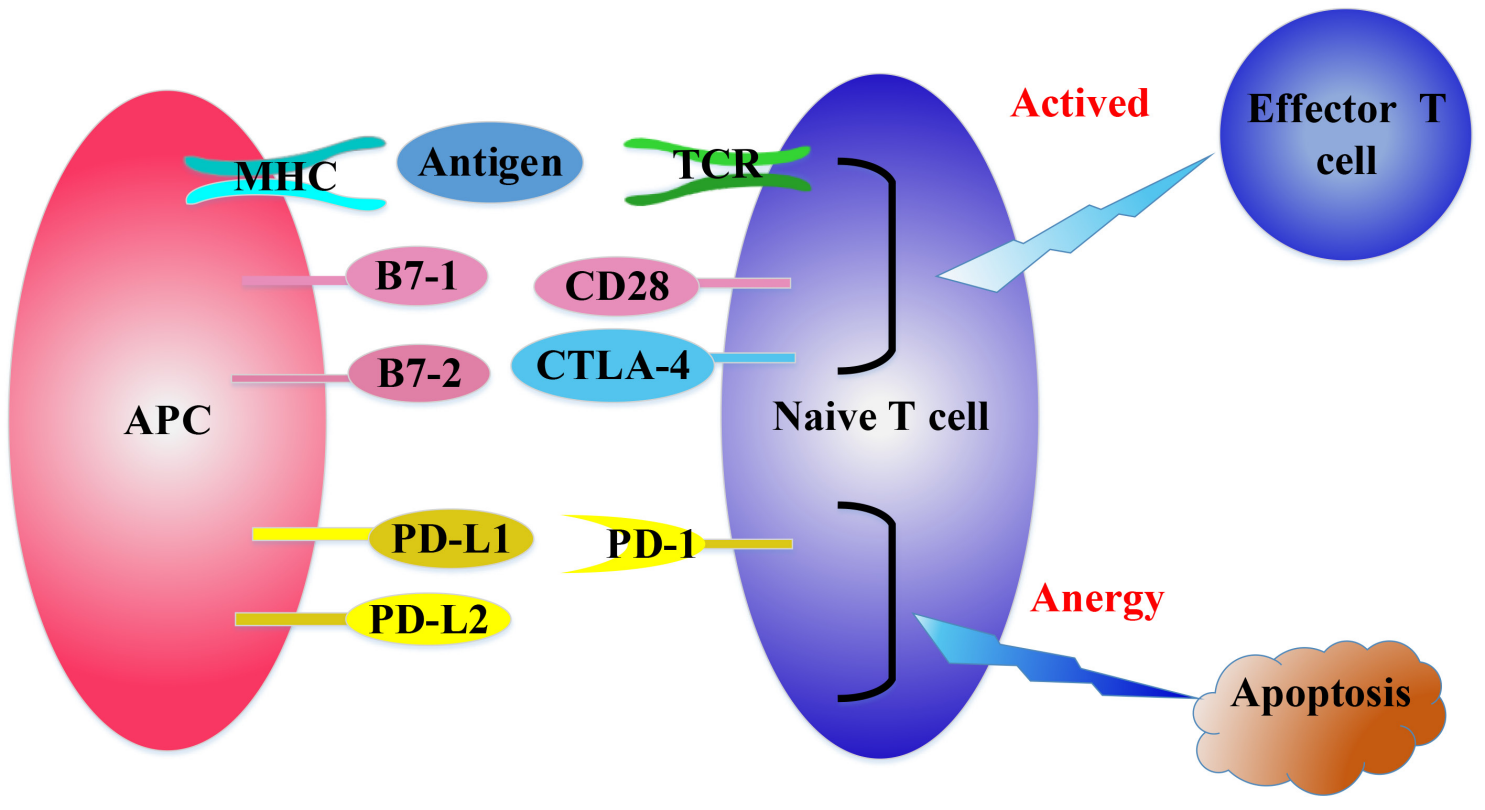
The regulation of T cell activation T cell receptor (TCR) recognizes the tumor antigens in the context of major histocompatibility complex (MHC) expressed on professional antigen presenting cells (APCs). Then APCs deliver a second signal by positive co-stimulatory molecules CD28/B7-1/B7-2 to fully activate naive T cells. CTLA-4 can bind with B7-1/B7-2 competitively to inhibit the activation of CD28/B7-1/B7-2, resulting in inactivation of T cells. PD-1 binds with its ligands, PD-L1 and PD-L2 to attenuate lymphocyte activation.

Immunotherapy is a rising hope for cancer patients, utilizing the immune system to detect and eliminate foreign tumor antigens. But immune response is such a complex phenomenon involving clonal T cell selection, activation, proliferation and trafficking to antigen sites to deliver immune effector functions, that it's hard to hit the target in immunotherapy [1]. The process of T cell activation requires two major signals (Figure 1): the co-stimulatory signals and co-inhibitory signals [2]. The co-inhibitory signals could be a main actor in cancer progression through the inhibition of anti-cancer immune response [3]. One of the inhibitory signals is PD-1/PD-L1 axis. PD-1 is a member of the B7 receptor family and is inducibly expressed on activated T cell subsets including T follicular helper (Tfh) cells [5] and T regulatory (Treg) cells. It attenuates immune responses by negatively regulating T cell proliferation and function [4]. And the relationship between PD-L1 expression on tumor and/or immune cells and objective immune response has been reported [6–9].

Chemotherapy is a conventional treatment for cancer with different extent of cytotoxicity but has immune potentiating mechanisms of action [10]. Whether chemotherapy can stimulate immune response and finally kill tumors is unknown. Recent introduction of immune modulators, PD-1/PD-L1 adds much excitement to this field. It is reported that the expression rate of PD-L1 in human malignant tumors varies from 19% to 92% [11] and the expression of PD-L1 is positively correlated with tumor progression [12–15]. PD-L1 overexpression predicted better pathological response to chemotherapy, independently of histo-clinical variables and predictive gene expression signatures [16]. Zhang *et al.* [18] demonstrated that paclitaxel, etoposide and 5-fluorouracil were able to induce PD-L1 surface expression in human breast cancer cells and increase PD-L1-mediated T cell apoptosis, revealing a potential link between chemotherapy and cancer immunoresistance. PD-L1 is expressed by cancer cells, the exact mechanism of how the chemotherapeutic drugs work on tumor microenvironment especially PD-1/PD-L1 axis and how this PD-1/PD-L1 axis induces chemoresistance is not clear. Herein, in this review, we try to summarize the relationships between chemotherapy and immune response through PD-1/PD-L1 axis.

## DIFFERENT CHEMOTHERAPEUTIC AGENTS HAVE DIFFERENT EFFECTS ON IMMUNE SYSTEM

**Figure 2:**
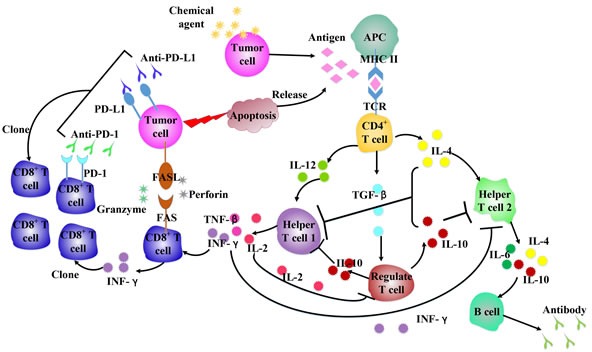
Chemotherapeutic agents influence cytokines network in antitumor immune system Different chemical agents work on immune cells, leading to various cytokines released, which can affect the immune cells populations to enhance/attenuate antitumor response.

Accumulating evidences suggest that conventional therapeutic regimens as well as targeted anticancer agents, originate (at least in part) from their ability to elicit a novel or reinstate a pre-existing tumor-specific immune response [19, 20]. One of the mechanisms is that chemotherapy can provoke the immune system to recognize and destroy malignant cells called immunogenic cell death (ICD) [21]. Several common chemotherapeutics share the ability to trigger ICD, (e.g., doxorubicin, epirubicin, idarubicin, mitoxantrone, bleomycin, bortezomib, cyclophosphamide and oxaliplatin) [21, 22] as well as some anticancer agents that are still under preclinical or clinical development (e.g., some microtubular inhibitors of the epothilone family) [21, 22]. Among the various chemotherapeutic drugs that have been tested on mice, anthracyclines are the only agents that provide enhanced immunity to further battle with tumor cells [23]. Recent data indicate that cyclophosphamide at high doses have the immunosuppressive properties, while metronomic cyclophosphamide regimens exert contrary immunostimulatory effects [20] by selectively depleting or inhibiting Tregs [24]. Such immunostimulatory properties seem to, at least in part, contribute to the therapeutic success by cyclophosphamide as a conventional anticancer agent [25]. Importantly, many clinical studies demonstrated that metronomic cyclophosphamide led to improved T cell effector functions [19, 26]. Cancer cells evade immune recognition via down-regulating human leukocyte antigen (HLA) Class I expression, allowing their escape from immune surveillance and destruction [27]. While in ovarian cancer cells, low-dose epothilone B, taxol and vinblastine greatly increased expression of HLA Class I and HLA-A2 molecules, and low-dose epothilone B treatment markedly increased the expression of interferon-α, IL-1β, IL-12 and IL-6 [27]. In the inflammatory microenvironment, interferon-γ (IFN-γ) and other inflammatory cytokines, secreted by anti-tumor Th1 cells or macrophages, may upregulate PD-L1 expression in response to immune-mediated attack [6], to decrease the cytotoxic local immune response. Some anti-tumor drugs can promote the cytokines (IFN, IL-6) release to upregulate PD-L1 constitutively or in response to inflammation [6]. PD-L1 is upregulated in cancerous cells *in vitro* by immune cytokines that are critical for T cell functioning, such as IFN-γ [28], which may even positively feedback to enhance immune tolerance *in vivo* (Figure 2). Collectively, these studies indicate that different chemotherapeutic agents have different effects on immune system.

## CHEMOTHERAPY ALTERS THE EXPRESSION OF PD-1/PD-L1

Besides inducing ICD, oxaliplatin are reported to inhibit the expression of programmed death ligand 2 (PD-L2), thereby limiting immunosuppression by both dendritic cells (DCs) and tumor cells [29]. Treatment with paclitaxel and etoposide upregulated PD-L1 expression in breast cancer cells, resulting in co-inhibitory signals activation [18]. Yang *et al.* [30] observed an increase of PD-L1 and PD-1 antigen expression in leukemia cells with decitabine treatment, and both PD-L1 and PD-1 expression were increased in a concentration dependent manner. QIN *et al.* [31] demonstrated that when the cisplatin concentration is less than IC_50_, cisplatin could upregulate PD-L1 expression in hepatoma H22 cells. Meanwhile, cisplatin could activate the phosphorylation of ERK1/2, and that cisplatin-induced PD-L1 expression is dependent of ERK1/2 phosphorylation [31]. Oki *et al.* [32] observed a suppression of PD-1 expression after treatment with panobinostat (a histone deacetylase). It suggests that panobinostat may exert anti-tumor activity by decreasing PD-1 expression in normal lymphocytes, stimulating the immune reaction against lymphoma [32]. PD-L1 and its signaling pathway appear to be a potential therapeutic target for cancer. Interestingly, a recent research demonstrated that PD-L1 expression had the capability to change over time with anti-PD-L1 antibody therapy [33]. Therefore, its expression status of a specific tumor tissue may not reflect the present immunologic phenotype of tumor. Doxorubicin is reported to downregulate PD-L1 expression on cell surface, while upregulate its nuclear expression in breast cancer cells [34]. A decrease in PD-L1 expression on cell surface is expected to increase the immunogenicity of the cancer cells, and its translocation to the nucleus is likely to be responsible for the anti-apoptotic impact of anthracyclines on cancer cells and their microenvironment [34]. The translocation of PD-L1 from the cell surface to the nucleus induced by doxorubicin occurs concurrently with AKT phosphorylation, but the PI3K/AKT pathway is not involved in this process [34], which indicates that the PD-L1 re-distribution from the cell surface to the nucleus is regulated by two signaling, including an AKT-dependent pathway (dominant in the nucleus) and an unknown AKT-independent pathway (dominant on the cell surface). Moreover, doxorubicin combined with PD-L1 knockdown has been shown to enhance apoptosis [34]. This indicates that its nuclear localization can enhance the anti-apoptotic function, which may link with the apoptotic machinery of the cell. As for the molecular mechanism of how to regulate PD-L1 expression has yet to be understood, but several researchers described the presence of pro-inflammation and inflammation may take part in it [35]. Further studies are needed to explore the mechanism of chemotherapy-induced PD-L1 expression in cancer cells.

## SOME SIGNAL MOLECULES ASSOCIATED WITH PD-L1 EXPRESSION

**Figure 3:**
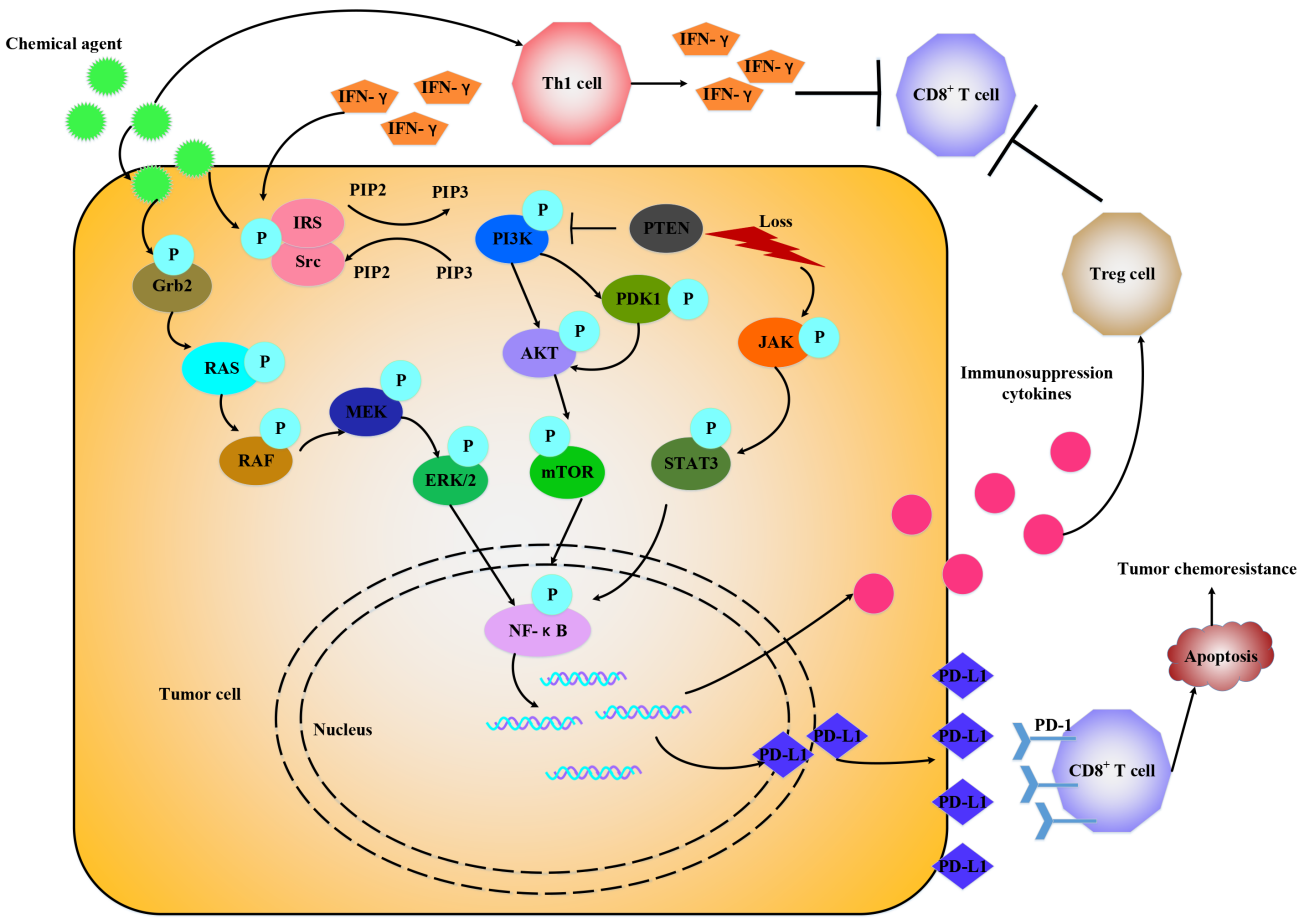
Chemotherapeutic agents promote PD-1/PD-L1 expression through various signals Chemotherapeutic agents *via* IFN-γ-dependent and IFN-γ-independent way to upregulate PD-L1 expression by activating different signals, like RAS/RAF, PI3K/AKT, JAK/STAT3 and release some immune suppression cytokines to attenuate antitumor immune response.

Zhang *et al.* [18] showed that chemotherapeutic agents potentiated IFN-γ-induced PD-L1 expression in human breast cancer cells. All the chemotherapeutic agents tested in the study had similar effects on PD-L1 surface expression in breast cancer cells [18], suggesting that they may act through a common pathway (Figure 3). IFN-γ enhances cancer immunoresistance by upregulating the expression of PD-L1 and PKD2 (Polycystic Kidney Disease Gene 2) in human oral squamous carcinoma cells in both time and dose dependent manner [36]. PKD2 knockdown with shRNA / siRNA or PKD chemical inhibitor resulted in IFN-γ production, then downregulated the expression of PD-L1 [36]. And the activation of PKD2 can stimulate the expression of P glycoprotein (P-gp) [37]. Inhibition of PKD2 activation could significantly inhibit the expression of P-gp and decrease multiple drug resistance (MDR) in human breast cancer cells [38], indicating that PKD2 may be an important target for tumor biotherapy and MDR reversal. Signaling through key proliferative pathways, like MEK/ERK and PI3K/AKT can also increase PD-L1 expression in malignant glioma, prostate and breast carcinoma [39, 40]. Berthon *et al.* [41] confirmed that blocking MEK inhibited PD-L1 transcription in the AML cell lines THP-1 and U937, suggesting that MEK is an important regulator of PD-L1 expression in leukemic cells. Non-small-cell lung cancer (NSCLC) cell lines bearing EGFR, KRAS, BRAF, ALK or RET mutations were found with high level of PD-L1 expression, and this may be correlated with high levels of PI3K/AKT/mTOR pathway activation. PD-L1 expression markedly increased in a subset of patients after acquiring resistance to gefitinib in EGFR-mutant NSCLC [42]. Loss of the phosphatase and tensin homolog (PTEN) through genetic deletions or mutations accelerates PD-L1 expression in tumors [43]. Crane *et al.* [40] demonstrated that PI3K activation caused by loss of PTEN function enhanced PD-L1 protein level expression in breast cancer cell lines. Parsa *et al.* [43] found an increase of the post-transcriptional PD-L1 expression in other types of PTEN loss cancers with the activation of the PI3K pathway. The transformed cells can also utilize PI3K pathway to evade the immune system by mimicking immune cells [44], developing resistance to T cell induced apoptosis [45], secreting immunosuppressive cytokines [46], enhancing the immunosuppressive potential of Treg cells [47] or emulating immune cell chemotactic responses [48]. Inhibition of PI3K or its downstream signaling molecule AKT decreased PD-L1 expression in tumor cells and increased cytotoxic T cells-induced killing [43]. Therefore, targeting PD-1/PD-L1 interaction may be enhanced when combined with PI3K inhibitors. The complex interaction between PI3K signal and anti-tumor immune response needs further studies. It also represents a promising avenue to best exploit the anti-tumor effects of clinical PI3K inhibitors. Toso *et al.* [49] showed that in PTEN-null senescent tumors, activation of the JAK2/STAT3 pathway induced an immunosuppressive tumor microenvironment that contributed to tumor growth and chemoresistance. Inhibition of the JAK2/STAT3 pathway in PTEN-deficient prostate tumors led to senescence-associated cytokine network reprogrammed, and improved the efficacy of docetaxel-induced senescence by triggering a strong anti-tumor immune response. Soliman *et al.* [37] reported that high PD-L1 basal cell lines had lower expression of IRF2BP2 (interferon regulatory factor 2 binding protein 2) and higher STAT1 levels compared to those with low PD-L1 expression. All findings above suggest that regulation of PD-L1 expression varies widely among cell types and drugs targeting signal transduction pathways might have different immunological effects in different tumors.

## PD-1/PD-L1 IN CHEMORESISTANCE

A research [41] showed that in five out of nine patients with AML, spontaneous PD-L1 expression increased when they relapsed. Lower expression of PD-L1 was positive correlated with a tendency to longer survival [30]. Jennifer *et al.* [50] found that in progressing prostate cancer patients, more PD-L1/2^+^ DCs led to poorer response to Enzalutamide (ENZ) treatment and shorter treatment duration. They also observed that circulating PD-L1/2^+^ DCs significantly increased in mice bearing Enzalutamide resistant (ENZR) tumors compared to castration resistant prostate cancer, and ENZR tumors expressed significantly increasing levels of tumor-intrinsic PD-L1. Altogether, it suggests that there are high expressions of PD-1/PD-L1 pathway molecules in peripheral blood immune cells in patients with ENZR castration resistant prostate cancer (CRPC). Another research [51] showed that drug-resistant osteosarcoma cell line KHOSR2 and virally-derived osteoblast cell line hFOB had high (3-log) PD-L1 gene expression, and osteosarcoma cell line SaOS and breast cancer cell line MCF-7 had low (< 1-log) expression. Each osteosarcoma cell line generally has various PD-L1 expression, ranged from low to high PD-L1 expression, with slightly higher expression from drug-resistant variants (KHOSR2 and U-2OSTR) than their parental cell lines (KHOS and U-2OS) [51]. Thus, PD-1/PD-L1 blockade has the potential to overcome resistance, and the combination therapy of chemotherapy and PD-1/PD-L1 blockade has the potent synergistic effects to enhance antitumor immunity. Schatton *et al.* [52] identified tumorigenic human ABCB5^+^ MMICs (a novel type of cancer stem cells, malignant melanoma-initiating cells), expressing chemoresistance determinant ABCB5 preferentially expressed PD-1 and B7-2, but with downregulated expression of PD-L1 compared to ABCB5^−^ cells [53]. The relationship between PD-L1 and chemoresistance determinant ABCB5 is worthy to further study. The PD-L1^high^ cells were demonstrated significantly resistant to CDDP (compound Danshen dripping pills) and TXL (Tongxinluo) compared with the PD-L1^low^ cells [54]. As for the mechanism of how PD-L1 increases the chemoresistance, Yu Fujita *et al.* [54] found that a miR-197 mimic can sensitize PD-L1^high^ drug-resistant cells to chemotherapy, indicating that the biological interaction between PD-L1 and chemoresistance occurs through the microRNA regulatory cascade. The overexpression of miR-197 induced decreased expression of PD-L1 in NSCLC cells [54]. The miR-197/CKS1B/STAT3 axis can drive tumor PD-L1 expression as a biomarker of this cascade, and miR-197 replacement therapy may be a potential treatment strategy for chemoresistant NSCLC [54].

## STRATEGIES FOR BLOCKING PD-1/PD-L1 AXIS IN CHEMORESISTANCE

**Figure 4:**
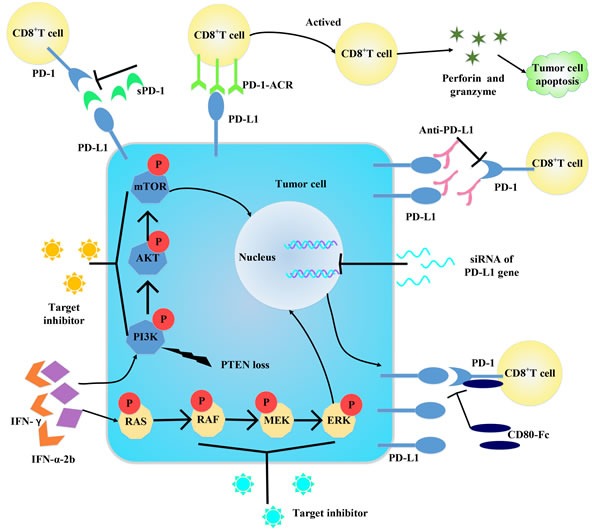
Some strategies for blocking PD-1/PD-L1 axis Targeting PD-1/PD-L1 and its downstream molecules can block the axis to activate. And siRNA can downregulate PD-L1 expression from gene level. Changing the construct of PD-1 can convert it into a co-stimulatory molecule to enhance immune response. Target IFN-γ can decrease IFN-γ-induced PD-L1 expression. Administration of molecules bind with PD-1/PD-L1 can interfere the binding between PD-1 and PD-L1, thus inactive the PD-1/PD-L1 axis.

With its profound immunosuppressive effect, PD-1/PD-L1 axis has been the focus of several recent studies aiming at neutralizing its detrimental effects on T cell anti-tumor response (Figure 4). There are now multiple agents targeting the PD-1/PD-L1 at different stages of clinical development [9, 55–57] (Table 1). PD-1 and PD-L1 antibodies have shown considerable clinical efficacy and durability across a range of malignancy subtypes, including melanoma and lung cancer [9, 58] and most recently in refractory Hodgkin's disease [59], and quite a lot of phase II studies are ongoing in prospect (NCT02572167, NCT02181738, NCT02327078). It decreases the metastatic risk and improves the therapeutic response when associated with immunogenic anti-cancer chemotherapy such as doxorubicin [60, 61]. As some signal molecules are reported to upregulate PD-L1 expression, the target inhibitors may be a potential treatment (Figure 4). Other immune modulatory agents, like IFN-α-2b, are going on clinical trials combined with different anti-PD-1 and anti-PD-L1 antibodies (NCT01943422, NCT01608594). Nivolumab (NCT02464657, NCT01658878), pembrolizumab (NCT02551432), MEDI4736 (NCT02027961), and MPDL3280A (NCT01633970, NCT02525757, NCT02409355) are being evaluated in combination with chemotherapies, tyrosine kinase inhibitors, or other targeted therapies. Recently, Tang *et al.* [62] converted PD-1 to a T cell co-stimulatory receptor by exchanging its trans-membrane and cytoplasmic tail with CD28 and 4-1BB signaling domains (PD-1-CD28-4-1BB, PD-1-ACR), which retained the ability to bind PD-L1, but resulting in T cell activation as evidenced by the elevated activity of PI3K/AKT, the augmentation of cytokine secretion and the increased proliferative capacity. Samuel *et al.* [63] reported that CD80-Fc (the fragment of CD80 IgG) is more effective in preventing PD-1/PD-L1-induced suppression and restoring T cell activation compared to treatment with mAb to either PD-1 or PD-L1. Soluble PD-1 (sPD-1) is an efficient way to bind PD-L1 and to block PD-1/PD-L1 interactions, in conjunction with a two-domain molecule of fibronectin (CH50) in inhibiting tumor invasion and growth in hepatoma [64]. A study [65] showed that using short-hairpin double-stranded silencing RNA (siRNA) to restrain the expression of PD-1 on the cell surface of tumor-specific T cells, improved immune responses. In addition, it remains to be seen whether ICD inducers (e.g., doxorubicin, epirubicin, etc.) may be advantageously combined with non-immunogenic conventional chemotherapeutics, targeted anticancer agents and/or immunostimulatory strategies. It is crucial for the discovery of next-generation chemotherapeutics, i.e., molecules that simultaneously hit cancer cells while exerting potent immunostimulatory effect. These agents may be particularly relevant for the development of combinatorial chemotherapeutic regimens that actively engage the host immune system against malignant cells.

**Table 1:** Some anti-PD-1/PD-L1 antibodies in clinical trials

Agents	Alias	Target	Clinical trial	Application	Details
Nilvolumab	BMS-936559, MDX-1106	PD-1	Approved by FDA	Melanoma, renal cell carcinoma and non-small cell lung cancer	/
Pembrolizumab	MK-3475, Lambrolizumab	PD-1	Approved by FDA	Advanced melanoma	/
Pidilizumab [66]	CT-011	PD-1	Phase II	Hematological malignant tumor	72 patients enrolled, PFS: 0.72 (90% CI, 0.60 to 0.82), toxicity: 23/72 (31.94%) blood and lymphatic system disorders
MPDL3280A	Atezolizumab	PD-L1	Phase III	Non-small cell lung cancer,	Ongoing (NCT01846416)
MEDI4736	Durvalumab	PD-L1	Phase II	Non-small cell lung cancer	Ongoing (NCT02087423)
Avelumab	MSB0010718C	PD-L1	Phase II	M-Merkel cell cancer	Ongoing (NCT02155647)

## SUMMARY

A better understanding of how chemotherapy affects the anti-tumor immunity and causes chemoresistance is crucial. But it is still unknown how the signaling events regulate the expression of these molecules in resistant cancer cells. Blockade of the PD-1/PD-L1 pathway is a new, promising immunotherapy for cancer. Strategic combination of immunotherapy and chemotherapy can effectively change the overall tumor microenvironment, as well as immune tolerance and immune suppression, which can maintain effective and durable anti-tumor immune response.
